# Identification of Traditional Chinese Medicine Constitutions and Physiological Indexes Risk Factors in Metabolic Syndrome: A Data Mining Approach

**DOI:** 10.1155/2019/1686205

**Published:** 2019-02-03

**Authors:** Yanchao Tang, Tong Zhao, Nian Huang, Wanfu Lin, Zhiying Luo, Changquan Ling

**Affiliations:** ^1^Department of Traditional Chinese Medicine, Changhai Hospital, The Second Military Medical University, Shanghai 200433, China; ^2^Department of Health Management, Navy District of Hangzhou Sanatorium of PLA, Hangzhou 310002, China

## Abstract

**Objective:**

In order to find the predictive indexes for metabolic syndrome (MS), a data mining method was used to identify significant physiological indexes and traditional Chinese medicine (TCM) constitutions.

**Methods:**

The annual health check-up data including physical examination data; biochemical tests and Constitution in Chinese Medicine Questionnaire (CCMQ) measurement data from 2014 to 2016 were screened according to the inclusion and exclusion criteria. A predictive matrix was established by the longitudinal data of three consecutive years. TreeNet machine learning algorithm was applied to build prediction model to uncover the dependence relationship between physiological indexes, TCM constitutions, and MS.

**Results:**

By model testing, the overall accuracy rate for prediction model by TreeNet was 73.23%. Top 12.31% individuals in test group (n=325) that have higher probability of having MS covered 23.68% MS patients, showing 0.92 times more risk of having MS than the general population. Importance of ranked top 15 was listed in descending order . The top 5 variables of great importance in MS prediction were TBIL difference between 2014 and 2015 (D_TBIL), TBIL in 2014 (TBIL 2014), LDL-C difference between 2014 and 2015 (D_LDL-C), CCMQ scores for balanced constitution in 2015 (balanced constitution 2015), and TCH in 2015 (TCH 2015). When D_TBIL was between 0 and 2, TBIL 2014 was between 10 and 15, D_LDL-C was above 19, balanced constitution 2015 was below 60, or TCH 2015 was above 5.7, the incidence of MS was higher. Furthermore, there were interactions between balanced constitution 2015 score and TBIL 2014 or D_LDL-C in MS prediction.

**Conclusion:**

Balanced constitution, TBIL, LDL-C, and TCH level can act as predictors for MS. The combination of TCM constitution and physiological indexes can give early warning to MS.

## 1. Introduction

Metabolic syndrome (MS) is a condition with a cluster of metabolic abnormalities that are characterized by central obesity, hypertension, hyperglycemia, and dyslipidemia [1]. The prevalence of MS is increasing rapidly worldwide [2, 3]. In China, the overall standardized prevalence of MS in adults is reported to be 24.2% and is increasing year by year due to the rapid economic growth [4]. According to the International Collaborative Study of Cardiovascular Disease in ASIA (InterASIA), the age-standardized prevalence of MS was 13.7% among adults aged 35-74 years in China between 2000 and 2001 [5]. Based on 2010 China Noncommunicable Disease Surveillance data assessed using National Cholesterol Education Program Adult Treatment Panel III (NCEP ATP III) criteria, the prevalence of MS among participants aged >/= 18 years was 33.9% [6].

MS is associated with an increased risk of diseases, such as cardiovascular disease (CVD), type 2 diabetes mellitus (DM), and cancer [7, 8]. Mottillo S. et al. conducted a meta-analysis containing 87 studies and found out that the metabolic syndrome was associated with an increased risk of CVD disease, CVD mortality, all-cause mortality, and myocardial infarction. Even without diabetes, MS patients maintained a high cardiovascular risk [9]. Therefore, the rapid diagnosis and prevention of MS are of great significance for the prevention of CVD and type 2 DM.

There are many studies on the etiology and influencing factors of MS [10, 11]. Researches showed that both innate and acquired factors were involved in the occurrence and development of MS [12]. It is consistent with the cognition of the diseases' occurrence and development in TCM constitution theory. In constitution theory of TCM, constitution was described as an integrated, metastable, and natural specialty in figure, physiological functions, and psychological conditions formed on the basis of innate and acquired endowments in the human life process [13]. In other words, constitution is a dynamic character with both nature and environment and is always developing in the process of human growth [14]. Constitutions in TCM are generally classified based on the physiological (e.g., blood flow, pulse, and heartbeat) and physical status (e.g., facial appearance and body figure), as well as the clinical characteristics [15, 16]. It was reported that some chronic diseases were closely related to biased constitutions [17–19]. Therefore, we hypothesized that the early imbalance trend of balanced constitution and the formation of biased constitution can predict the occurrence of metabolic syndrome together with physiological indexes.

Previous epidemiological studies of MS usually use multiple linear regression, logistic regression, or Cox regression models to screen risk factors. However, these methods are limited for strict requirements on data type, distribution and multicorrelation problems.

Data mining is the process of uncovering patterns, classifications, and relationships in large datasets using methods at the intersection of machine learning, statistics, and database systems [20]. The data mining process enables data owners to better understand the dependencies between the attributes of the data samples and predict the corresponding subsystem behavior.

TreeNet is a novel advance in data mining proposed by Friedman [21] at Stanford University. It builds trees from hundreds of small trees and each tree depicts a small portion of the overall model. The model prediction is finally done by adding up all individual contributions [22]. TreeNet is a new machine learning approach which is efficient for regression problems. TreeNet is fast data driven, immune to outliers and invariant to monotone transformation of variables. Therefore, TreeNet models inherit almost all of the advantages of tree-based models, while overcoming their primary disadvantages [23]. In this study, it was applied to identify TCM constitutions and physiological indexes that act as predictors for MS using two health datasets, providing support for the establishment of early warning system of MS.

## 2. Materials and Methods

### 2.1. Date Sources

**Figure 1:**
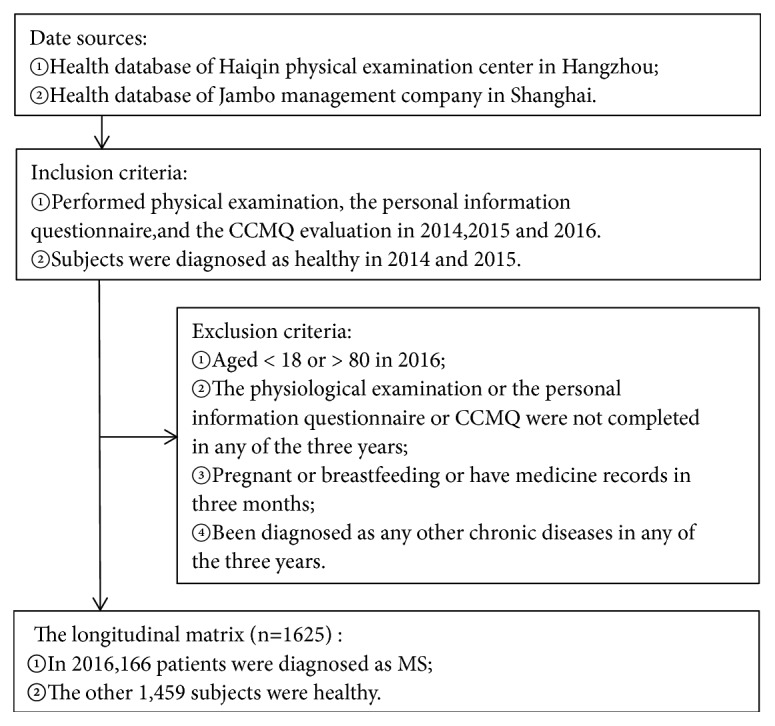
Flow chart of data filtering.

Use three consecutive years' data for model building. Qualified data was screened from health data from Hangzhou Haiqin Sanatorium and Shanghai Jambo Health Management Center. The data filtering process was shown in Figure 1.

A total of 1,625 individuals, including 1,037 men whose average age in 2014 was 40.01 ± 12.96 and 588 women whose average age was 41.29 ± 12.96, were finally qualified and entered into follow-up analysis. Usual place of residence of these individuals was mainly distributed in east and central China with 544 (33.48%) in Zhejiang province, 548 (33.72%) in Shanghai city, 222 (13.66%) in Jiangsu province, 53 (3.26%) in Fujian province, 122 (7.51%) in Hubei province, 135 (8.31%) in Shandong province, and 1 (0.62%) in Beijing city.

### 2.2. Evaluation Index and Outcome Measures

General information is name, sex, age, and native place.

Physiological indexes are (i) physical examination: height, weight, pulse, waist circumference (WC), systolic pressure (SBP), diastolic pressure (DBP), and Body Mass Index (BMI); (ii) biochemical tests: blood routine examination and biochemical indexes (direct bilirubin (DBIL), indirect bilirubin (IBIL), total bilirubin (TBIL), total protein, albumin, globulin, and albumin ratio, glutamic oxaloacetic transaminase (AST), glutamic-pyruvic transaminase (ALT) and AST ratio (AST/ALT), total cholesterol (TCH), triglycerides (TG), serum high-density lipoprotein cholesterol (HDL-C), serum low-density lipoprotein cholesterol (LDL-C), glucose (GLU), *γ*-glutamyltransferase (*γ*-GT), uric acid (UC), creatinine (Cr), urea nitrogen (BUN), tumor markers (carcinoembryonic antigen, alpha fetoprotein, ferritin, ca-199, ca-125, ca-153, prostate-specific antigen), and Thyroid function: T3, T4.

Constitution classification was as follows: CCMQ was used to investigate the constitution types of subjects. The assessment contains 5 aspects of measurement, including physical characteristics, psychological characteristics, reaction state, tendency to diseases, and adaptability. A total of 60 items were measured to classify a person into one or more of nine constitution types: balanced constitution (8 items), qi-deficient constitution (8 items), yang-deficient constitution (7 items), yin-deficient constitution (8 items), phlegm-dampness constitution (8 items), damp-heat constitution (6 items), stagnant blood constitution (7 items), stagnant qi constitution (7 items), and inherited special constitution (7 items).

MS identification was as follows: MS was identified according to the criteria set by Chinese diabetes society (CDS): (i) overweight and/or obesity: BMI ≥ 25.0 kg/m^2^; (ii) hyperglycemia: FPG ≥ 6.1 mmol/L and/or 2 hPG ≥ 7.8 mmol/L and/or treatment of previously diagnosed type 2 diabetes; (iii) hypertension: SBP ≥ 140 mmHg and/or DBP ≥ 90 mmHg and/or treatment of previously diagnosed hypertension; (iv) triglyceride abnormality: TG ≥ 1.7 mmol/L and/or low HDL-C (< 0.9 mmol/L for men, < 1.0 mmol/L for women). MS can be diagnosed if any 3 or all of the above conditions are met.

All the included indicators were analyzed, and the diagnostic results were confirmed by two or more doctors.

### 2.3. Data Cleaning

(i) The physical examination code was used as identification number of the subjects. (ii) Health data before 2014 was removed to avoid interference. (iii) Units and formats for data from different sources were uniformed. (iv) Converted scores were computed for each type of constitutions and used for analysis according to the scoring criteria of CCMQ. (v) Target status was as follows: in 2016, subjects who were diagnosed with MS were labeled 1 and healthy were labeled 0.

### 2.4. Procedure of TreeNet

In this study, the TreeNet models were constructed using TreeNet software by Salford Systems.

Parameter setting was as follows: (i) learn rate: auto; (ii) subsample fraction: 1.00; (iii) influence trimming factor: 0.10; (iv) M-regression breakdown: 0.99; (v) regression loss criterion: Huber-M.

Model construction was as follows: the model was begun with a small tree grew on original target and the residuals of this tree were computed. Then the second tree was built to predict the residual from the first tree. Next, we compute residuals from the new model of two trees, and a third tree was grown to predict revised residuals. We repeat the progress for machine learning and got a sequence of tree. At last, we added up all individual contributions.

### 2.5. Model Testing

The dataset was randomly categorized into two groups (a training group and a test group). The prediction model was developed on the basis of the training group which consisted of 1300 cases (80% of the entire dataset). Model validation was made on the basis of the test group consisting of the rest 20% of cases (325 cases).

## 3. Results

### 3.1. Accuracy of TreeNet Algorithms

Values of the area under the receiver operating characteristic (ROC) curve (AUC) were calculated to evaluate accuracy of the TreeNet model. AUC value for TreeNet was 0.694.

### 3.2. TreeNet Model Testing and Assessment

The confusion matrix for TreeNet algorithm was shown in Table 2, indicating that the model has certain predictability. Model validation was performed using the test group consisting of 20% of random data. In the test group, 287 individuals were diagnosed without MS, of which 219 were accurately predicted by the model, indicating the accuracy rate reached 76.31%. In addition, 19 of 38 patients diagnosed with MS were predicted by the model. The average accuracy rate was 63.15%, and the overall accuracy rate was 73.23% (Table 1).

The risk of MS of test group was graded according to the model. 325 cases were divided into 10 parts (10 bins) and ranked in Table 2. It was shown that the top 12.31% individuals that have a higher probability of having MS covered 23.68% MS patients at the bin of highest risk. In other words, this population was 1.92 times that of the general population at risk of having MS. Top 24% individuals covered 47.37% MS patients, showing 1.03 times more risk of MS than the general population. These results indicated high accuracy for true positive samples prediction.

### 3.3. Variables (Physiological Indexes or TCM Constitutions) Importance

TreeNet model gives stable variable importance rankings after assessing the relative importance of predictors. Importance of variables ranked top 15 was listed in descending order in Table 3. The top 5 were TBIL difference between 2014 and 2015 (D_TBIL); TBIL in 2014 (TBIL 2014); LDL-C difference between 2014 and 2015(D_LDL-C); CCMQ scores for balanced constitution in 2015 (balanced constitution 2015); and TCH in 2015 (TCH 2015).

**Figure 2:**
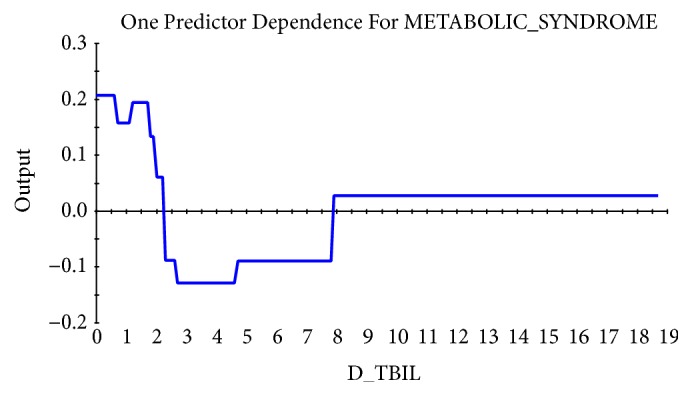
Dependency between TBIL difference between 2014 and 2015 (D_TBIL) and incidence of MS.

**Figure 3:**
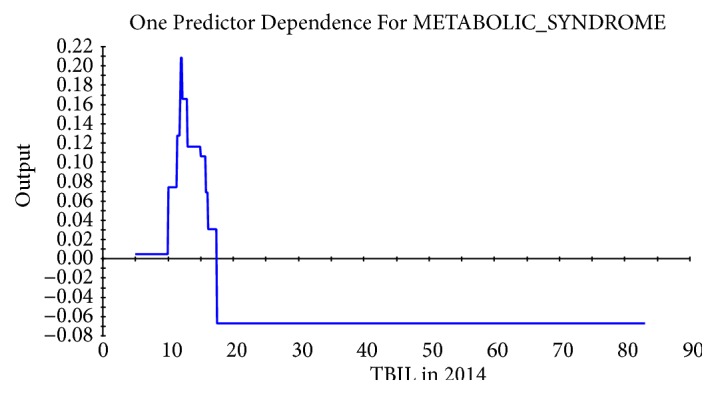
Dependency between TBIL in 2014 and incidence of MS.

**Figure 4:**
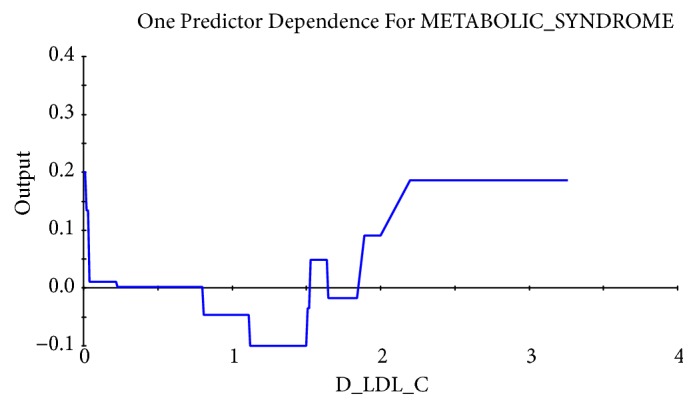
Dependency between LDL-c difference between 2014 and 2015 (D_LDL-c) and incidence of MS.

**Figure 5:**
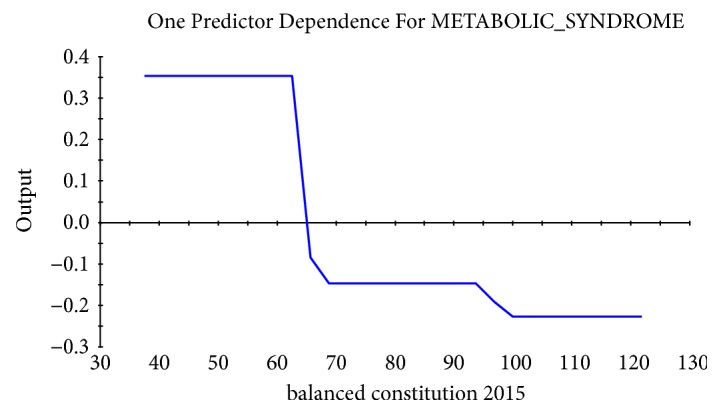
Dependency between CCMQ scores for balanced constitution in 2015 (balanced constitution 2015) and incidence of MS.

**Figure 6:**
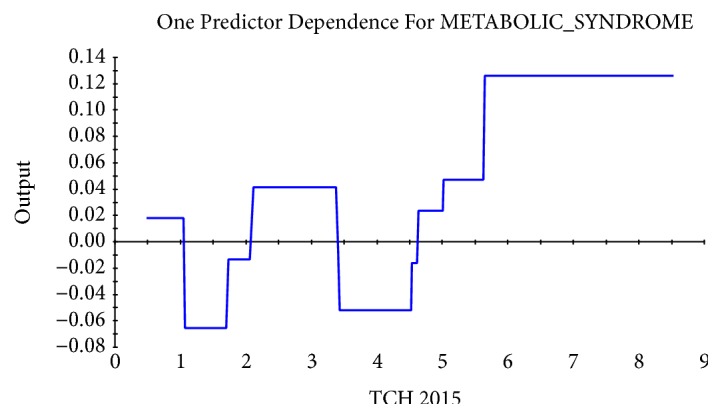
Dependency between TCH in 2015 (TCH 2015) and incidence of MS.

Taking the abscissa as the value of physiological indexes and the ordinate as the influence on the target, the relational dependency between D_TBIL, TBIL 2014, D_LDL-C, balanced constitution 2015, TCH 2015, and incidence of MS was shown in Figures 2–6. It came out that incidence of MS was higher when D_TBIL was between 0 and 2, TBIL 2014 was between 10 and 15, D_LDL-C was above 19, balanced constitution 2015 was below 60, or TCH 2015 was above 5.7.

### 3.4. Interaction of CCMQ Scores and Physiological Indexes in MS Prediction

**Figure 7:**
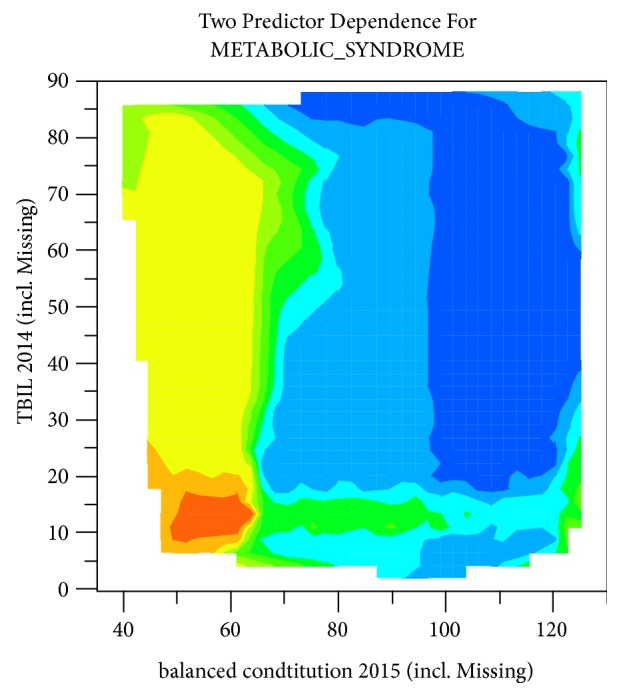
Interactive prediction with TBIL in 2014 (TBIL 2014) and CCMQ score for balanced constitution in 2015 (balanced constitution 2015).

**Figure 8:**
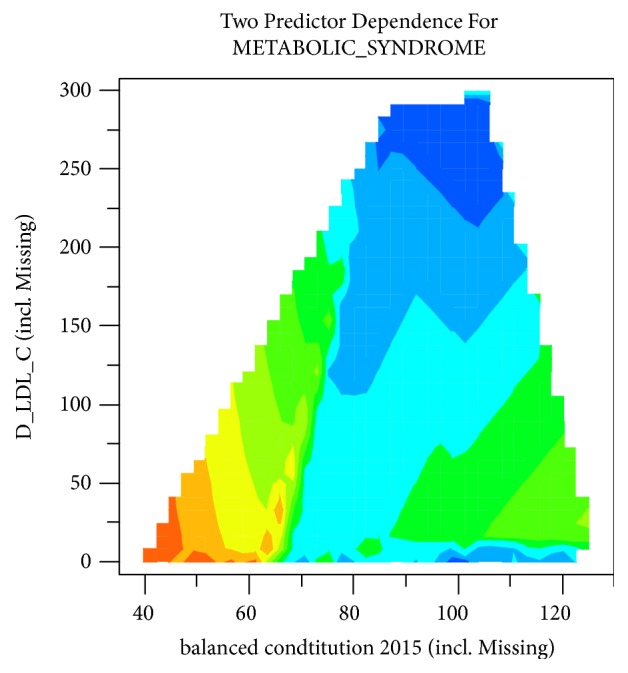
Interactive prediction with LDL-c difference between 2014 and 2015 (D_LDL-c) and CCMQ score for balanced constitution in 2015 (balanced constitution 2015).

Bivariate prediction considering pairwise interactions between CCMQ scores and physiological indexes was computed and displayed in heatmap format, with warm color representing positive correlation and cool color representing negative correlation. Two most significant ones were interactive prediction with TBIL 2014 and balanced constitution 2015 and interactive prediction with D_LDL-C and balanced constitution 2015 (Figures 7 and 8). It came out that the incidence rate of MS increased significantly in 2016 under the following two conditions: (i) balanced constitution 2015 score between 50 and 60 and TBIL 2014 between 8 and 17 and (ii) balanced constitution 2015 score below 60 and D_LDL-C below 47.

## 4. Discussion

Over the past decades, the metabolic syndrome prevalence has increased markedly worldwide, which may be explained by urbanization, an aging population, lifestyle change, and nutritional transition. Previous surveys indicated that metabolic syndrome has become a serious public health problem and highlights the urgent need to prevent and treat.

Chronic diseases were usually induced by both internal and external factors, such as genetic abnormalities, imbalance of intestinal flora, carcinogens, poor diet, and physical inactivity. Therefore, it is necessary to explore health parameters correlated with chronic diseases, for providing evidence for prediction and early diagnosis. Data mining technology has made great progress in disease prediction, diagnosis, and treatment for its advantage in analyzing data from a large pool of information to get knowledge of unknown patterns, classifications, clustering, and relationships [24–26]. TreeNet machine learning algorithm was used in exploring physiological parameters' change in MS development in our study by analyzing consecutive health data of subjects in 2014 to 2016.

Our results suggested that metabolic indexes as bilirubin and lipoprotein were important parameters to predict the occurrence of MS.

For a long time, bilirubin was considered as a waste. As the final product in catabolism of heme, bilirubin is often used as indicators in clinical diagnosis of hemolysis, neonatal jaundice, liver and biliary related diseases, etc. However, with the deepening of research, bilirubin is revealed to be a powerful antioxidant that suppresses the inflammatory process [27]. Besides, it was reported that bilirubin may be negatively correlated with MS [28]. This study explored the physiological predictors in individuals that were diagnosed as MS in 2016 for the first time or not. The results showed that the difference value of TBIL between 2014 and 2015 (D_TBIL) and TBIL in 2014 (TBIL 2014) can significantly predict the occurrence of MS in 2016. Especially when D_TBIL was 0-2 and/or TBIL 2014 was 10-15, the incidence of MS was the highest. Besides, consistent with researches before, the incidence of MS was decreased with increasing of the TBIL level, indicating that TBIL is expected to be a new predictive indicator in MS screening.

Lipid metabolism is closely related to MS. Lots of studies have confirmed that high-density lipoprotein, low-density lipoprotein, and cholesterol were positively correlated with MS [29]. Serum LDL-C level was reported to have a weak ability in predicting MS in women by analyzing the data from a population-based cross-sectional study conducted on representative samples of an Iranian adult population [30]. In present study, the risk of MS in 2016 was remarkably higher in individuals with the difference value of low-density lipoprotein cholesterol between 2014 and 2015 (D_LDL-C) above 19 and/or the total cholesterol in 2015 (TCH 2015) above 5.7.

Body constitution in traditional Chinese medicine is the fundamental physiological component of a person, and different constitution types are variously susceptible to diseases [31]. Previous studies have shown that biased constitutions were risk factors of chronic diseases. For example, yang-deficient constitution is an independent predictor of diabetic retinopathy in T2DM patients by multiple logistic regression analysis [32]. Yang-deficient and phlegm-dampness exhibited a significantly higher risk of albuminuria among T2DM patients [33]. These findings suggest that the constitution types found in people with chronic diseases may provide valuable information for disease prevention and treatment [34]. Our study found consistent results. In our study, TCM constitutions played an important role in the MS prediction. Balanced constitution 2015, Stagnant blood constitution 2015, D_yin-deficient constitution, D_balanced constitution, phlegm-dampness constitution 2015, Stagnant qi constitution 2014, D_stagnant blood constitution, and inherited special constitution 2015 can predict the occurrence of MS in 2016.

One notable difference from previous studies is that balanced constitution is more important than biased constitution in disease prediction. Balanced constitution is a “strong and robust physical state”. People of balanced constitution were in moderate shape with flushing complexion and were energetic. Grading criterion of balanced constitution is that converted score for balanced constitution is equal to or greater than 60 and converted scores for 8 other biased constitutions were less than 30. It is believed in traditional Chinese medicine that the body's state from “health” to “sick” is due to damaging of the body's balance state by internal and external causes. A converted score for balanced constitution less than 60 is a sign of the gradual weakness of balanced constitution and the formation of the biased constitutions. According to the prediction model in this study, when the CCMQ scores for balanced constitution in 2015 (balanced constitution 2015) were lower than 60, the prevalence of MS was higher. Furthermore, interaction of CCMQ scores and other physiological indexes in prediction of MS was analyzed. Results indicated that balanced constitution 2015 had an interaction with TBIL 2014 and D_LDL-C, and loss of balanced constitution combined with low level of TBIL in 2014 or D_LDL-C is important predictors of the occurrence of MS. It noted that the change of balanced constitution may be an earlier indicator that can predict the trend of morbidity and must be taken seriously in clinic.

This research is the first to build forecasting model by data mining method to explore prediction effect of TCM constitutions and other physiological parameters on MS incidence by analyzing consecutive health data of subjects. It provides evidence that the physiologic indexes and TCM constitutions can provide predictive information before the occurrence of MS. Maintaining CCMQ scores of balanced constitution higher than 60 points and reasonable levels of TBIL, LDL-C, and TCH can help to delay the occurrence of MS.

## Figures and Tables

**Table 1:** The confusion matrix.

Actual Class	Total Class	Percent Correct	Predicted Classes	
			0	1
			N=238	N=87
0	287	76.31%	219	68
1	38	50.00%	19	19
Total	325			
Average		63.15%		
Overall % Correct		73.23%		

**Table 2:** Yield of prediction model (N=325).

Rank	Cases in each bin	Percentage of test cases (n=325)	Cumulative percentage of test cases	Diagnosed cases in each bin	MS prevalence in each bin	Percentage of total diagnosed cases (n=38)	Cumulative percentage of total diagnosed cases (n=38)	Lift (times)
1	40	12.31	12.31	9	22.50	23.68	23.68	1.92
2	38	11.69	24.00	9	23.68	23.68	47.37	2.03
3	36	11.08	35.08	4	11.11	10.53	57.89	0.95
4	34	10.46	45.54	5	14.71	13.16	71.05	1.26
5	34	10.46	56.00	3	8.82	7.89	78.95	0.75
6	32	9.85	65.85	3	9.38	7.89	86.84	0.80
7	30	9.23	75.08	2	6.67	5.26	92.11	0.57
8	28	8.62	83.69	2	7.14	5.26	97.37	0.61
9	27	8.31	92.00	1	3.70	2.63	100	0.32
10	26	8.00	100	0	0.00	0.00	100	0

**Table 3:** Variables of high predictive value. (Prefix D_notated difference between 2014 and 2015).

rank	Variable	Score
1	D_TBIL	100
2	TBIL 2014	94.88
3	D_LDL-C	91.74
4	Balanced constitution 2015	88.55
5	TCH 2015	87.91
6	ALT 2014	87.38
7	ALT 2015	86.46
8	T3 2015	82.79
9	D_BUN	78.78
10	Stagnant blood constitution 2015	73.05
11	D_yin-deficient constitution	73.01
12	D_ALT	72.95
13	D_TCH	71.87
14	D_*γ*-GT	70.88
15	D_balanced constitution	65.38
16	*γ*_GT 2015	63.24
17	*γ*_GT 2014	59.65
18	Phlegm-dampness constitution 2015	50.72
19	Stagnant qi constitution 2014	48.24
20	D_stagnant blood constitution	48.08
21	Inherited special constitution 2015	43.56
22	BUN 2015	42.14
23	BUN 2014	26.57

## Data Availability

Due to the sensitive nature of the questions asked in the Constitution in Chinese Medicine Questionnaire (CCMQ), survey respondents were assured raw data would remain confidential and would not be shared. All cleaned data we used in the article are transparent and available upon request by contact with the corresponding author.
